# Public Perception of SARS-CoV-2 Vaccinations on Social Media: Questionnaire and Sentiment Analysis

**DOI:** 10.3390/ijerph182413028

**Published:** 2021-12-10

**Authors:** Charlotte Roe, Madison Lowe, Benjamin Williams, Clare Miller

**Affiliations:** 1 School of Life Sciences, University of Lincoln, Lincoln LN6 7TS, UK; 15568465@students.lincoln.ac.uk (C.R.); 16655259@students.lincoln.ac.uk (M.L.); 2 School of Computer Science, University of Lincoln, Lincoln LN6 7TS, UK; bWilliams@lincoln.ac.uk

**Keywords:** SARS-CoV-2, COVID-19, vaccinations, sentiment analysis, Twitter, anti-vax, vaccine hesitancy, Python, VADER, NLTK

## Abstract

Vaccine hesitancy is an ongoing concern, presenting a major threat to global health. SARS-CoV-2 COVID-19 vaccinations are no exception as misinformation began to circulate on social media early in their development. Twitter’s Application Programming Interface (API) for Python was used to collect 137,781 tweets between 1 July 2021 and 21 July 2021 using 43 search terms relating to COVID-19 vaccines. Tweets were analysed for sentiment using Microsoft Azure (a machine learning approach) and the VADER sentiment analysis model (a lexicon-based approach), where the Natural Language Processing Toolkit (NLTK) assessed whether tweets represented positive, negative or neutral opinions. The majority of tweets were found to be negative in sentiment (53,899), followed by positive (53,071) and neutral (30,811). The negative tweets displayed a higher intensity of sentiment than positive tweets. A questionnaire was distributed and analysis found that individuals with full vaccination histories were less concerned about receiving and were more likely to accept the vaccine. Overall, we determined that this sentiment-based approach is useful to establish levels of vaccine hesitancy in the general public and, alongside the questionnaire, suggests strategies to combat specific concerns and misinformation.

## 1. Introduction

### 1.1. Coronavirus Disease 2019 in the UK and Vaccination Uptake

Coronavirus disease 2019 (COVID-19), caused by novel severe acute respiratory Coronavirus 2 (SARS-CoV-2), was first reported in Wuhan, China in December 2019. As has already been well reported, COVID-19spread rapidly across the globe and was declared a pandemic by the World Health Organisation (WHO) in March 2020. In late January 2020, the first case was reported in the United Kingdom (UK) and by the end of March 2020, 6650 cases had been recorded in the UK and a nationwide lockdown had begun [1].

On 8 December 2020, the UK became the first country to rollout a COVID-19 vaccination programme; and by 15 August 2021, an estimated 87.1% of the adult population in the UK had received one dose of either the Oxford/AstraZeneca, Moderna or Pfizer-BioNTech vaccine and 74.9% were fully vaccinated with two doses [2]. Even before the first dose was administered, false rumours and misinformation had begun to circulate on social media, at times fuelled by the idea that emergency regulatory approval of these vaccines was linked to unreliability or safety concerns, threatening to diminish public confidence in the vaccination programme [3]. By 15 August 2021, the cumulative total of deaths in the UK where the death certificate mentioned COVID-19 as one of the causes was 157,361. The cumulative total number of doses of vaccinations administered in the UK on the same date was 88,037,283 [2].

### 1.2. Anti-Vaccination Movement

Since their introduction, vaccinations have revolutionised health care whilst at the same time persistently facing opposition [4,5] from hesitant individuals who perceive them as unnecessary or dangerous [6]. ‘Anti-vaccinators’ or ‘anti-vaxxers’ may reject vaccinations in the belief that they contain toxins and cause serious adverse effects [7]. More extreme conspiracy theories accuse pharmaceutical companies of producing fake vaccine data, concealing harmful vaccine side effects and exaggerating vaccine efficacy statistics [8].

Hesitancy is typically associated with a lack of trust in the health-care system [9] and unfamiliarity with vaccine-preventable diseases [10]. For example, in 1974, it was reported that an antigen in the pertussis vaccine was responsible for 36 neurological complications including convulsions and intellectual developmental disorders in previously healthy children. Despite the study concluding that these complications were extremely rare and the risks of immunisation outweighed the risks of disease [9], many parents in Britain refused to vaccinate their children against pertussis throughout the 1970s and 1980s. Between 1971 and 1974, vaccination rates dropped significantly from 78.5% to 37% [11], leading to severe strain on the NHS [12,13].

The measles, mumps, and rubella (MMR) controversy was the result of a now discredited paper linking the MMR vaccine to autism in children [8,14], which led to a reduction in MMR uptake after its publication in 1998 and the debate still rumbles on. Although MMR vaccination uptake has improved since 2004, according to the WHO, it is still under the 95% threshold to ensure herd immunity; and in 2017, an estimated 142,000 people died from measles unnecessarily [6,15,16], leading the WHO to declare vaccine hesitancy as an official threat to global health in 2019 [17] and highlighting the need for medical professionals to address vaccine safety concerns to encourage uptake.

### 1.3. Social Media and Vaccine Hesitancy

Web 2.0 has made discovering and sharing information online more convenient than ever with the move from passive consumption to active generation of content, leading to Health 2.0, where social media users share advice and experiences relating to health care [18]. However, despite social media being readily utilised to promote public health, and increasing numbers of people using social media to research vaccinations [17,19], health-care professionals remain a key source of vaccine information [20]. Media and celebrity opinion on social media is known to contribute to anti-vaccine beliefs [21] and the way in which research is interpreted by the media can have a profound effect on influencing public perception [22,23]. Scientists regularly challenge inaccurate information on social media and one high-profile example of this occurred in September 2021, when Professor Chris Whitty, the Chief Medical Officer for England and Chief Medical Advisor for the UK Government, was asked at a televised press conference about a tweet by rapper Nicki Minaj which claimed that her cousin’s friend was rendered impotent after taking a Coronavirus vaccine which caused swelling in his testicles. Prof Whitty said that these “myths… untrue… designed to scare… they should be ashamed”, leading to a conversation which continued afterwards in the media, including on social media. Despite progress being made to combat false reporting of science [23], understanding reasons behind vaccine hesitation will allow insight into how these beliefs may be counteracted effectively. Analysis of tweets during a 2013 measles outbreak [24] noted users informing each other about the importance of vaccination in light of the outbreak, illustrating a positive application of social media to educate others regarding the importance of vaccines to prevent outbreaks of disease.

However, the echo-chamber effect described by Piedrahita-Valdés et al. (2021), explains how users with differing beliefs consume homogeneously polarised content regarding vaccines and form opposing groups who rarely communicate with one another positively [25]. Hence, debate regarding vaccines may have little positive outcome, as prior personal beliefs are only reinforced in this environment. Efforts by health professionals to promote vaccination through social media have not always received a positive response; and in extreme cases, health-care professionals have been threatened after posting videos online encouraging vaccination [26].

During the UK national lockdowns in 2020 and 2021, much of the conversation regarding COVID-19 took place on social media platforms including Twitter, which has approximately 300 million monthly users [27,28]. Social media has become a common platform for individuals to voice their concern and share their thoughts with others during times of crisis [29]; but whilst these platforms allow the rapid dissemination of information, there is no guarantee that the information is correct, reliable or accurate [30] and the majority of anti-vaccination communication and conversation takes place over the internet [31]. Google search interest for the term ‘vaccine’ has greatly increased since March 2020, peaking in March 2021 [32].

In a July 2020 UK survey, 16% of participants stated that they would be unlikely to accept a COVID-19 vaccine [33]; and between September and October 2020, 12% and 17% of individuals were strongly hesitant or very unsure, respectively [34]. The likelihood of refusal of the COVID-19 vaccine was also found to be higher among young adults who are indifferent about COVID-19 and lack trust in scientists [33].

### 1.4. Sentiment Analysis and Data Mining

Natural language processing (NLP) research topics rely heavily on the use of sentiment analysis and opinion mining, where sentiment analysis is the study of opinions, feelings and attitudes towards a product, organisation or event [35,36,37]. Opinion—or text—mining involves extracting knowledge and information from online text, usually focusing on a certain topic and categorising it as positive, negative or neutral [38,39].

Python is a versatile computer programming language which can manage large datasets, making it ideal for use in complex projects [40,41,42]. It can be used to retrieve tweets that contain chosen search terms and store them via a designated database engine, such as SQLite. Valance Aware Dictionary and sEntiment Reasoner (VADER) is one of many tools found within the popular Natural Language Toolkit (NLTK), with an excess of 9000 lexicon features and the ability to analyse sentiments extracted from social media sources. It produces a gold-standard sentiment lexicon by combining quantitative and qualitative methods [43]. Sentiment lexicons contain lists with initial lexical capabilities (words) categorised to a semantic orientation (i.e., positive or negative) [38,44]. The VADER lexicon is a collection of predefined words with an associated polarity score—analysing the positive and negative aspects of text and determining overall polarity. Typically, neutral sentiments have a polarity score of 0 due to unidentifiable sentiment in the text. Negative and positive sentiments are assigned polarity scores of less than and greater than 0, respectively [45]. According to Satter et al. (2021), it is one of the easiest approaches to sentiment classification [28] with VADER based on a gold-standard sentiment lexicon with an ability to process acronyms and slang words [46], making it highly sensitive to sentiment expressions when applied to social media contexts. Hutto and Gilbert (2014) determined that VADER analysis performed better in comparison to eleven other highly regarded sentiment models and interestingly the accuracy of VADER has been determined to outperform individual human analysers at correctly classifying the sentiment of tweets [47]. In the majority of machine learning approaches to sentiment classification, for example, Microsoft Azure’s Text Analytics suite, a labelled dataset is required, whereby the polarity of text is predefined. Whilst Azure’s graphical interface can be utilised by individuals with little to no formal computer programming experience, making it an ideal software to use for novices, VADER, on the other hand, requires domain-specific knowledge of computing to use.

### 1.5. Sentiment Analysis of Vaccine Hesitance

Vaccine hesitancy is a fluid and ever-changing phenomenon [47]. Previous studies have typically focused on vaccine hesitance in general rather than being directed at specific vaccines and have revealed different trends across time [25,48]. Rahim et al. (2020) analysed approximately 100,000 tweets about vaccinations between October 2019 and March 2020 and determined that the majority (41%) were positive in sentiment, closely followed by neutral sentiment (39%) and 20% were negative [48]. COVID-19-specific vaccine hesitancy has also been investigated: in May 2020, vaccine hesitancy rates were low (20–25%) in American and Canadian adults [49], whereas, in Italy, the rates of COVID-19 vaccine hesitancy were 41% [50] and 26% in France [51].

### 1.6. Research Involving Questionnaires

Before the explosion of online sentiment mining, researchers solely used qualitative data collection methods in the form of surveys and particularly questionnaires [52]. Online questionnaires have many advantages, including increased collection of data, decreased cost and time to collect data and readily exportable formats for analytical simplicity [53,54]. To establish trends, attitudes and patterns, questionnaires are usually incorporated into mixed-method research and often yield information that computer-based programs may not identify. For example, questionnaires can extract demographic information and include questions exploring the reasoning behind opinions [54].

### 1.7. Aims and Objectives

The overall aim of this study was to determine the sentiment of public opinion regarding COVID-19 vaccinations. This was carried out via sentiment analysis of English language tweets on Twitter and followed up with a questionnaire which was distributed from the UK. The goal of the questionnaire was to explore attitudes to the expression of any particular sentiment, rather than to find any specific correlation between the two. Specifically, we aimed to determine the following:

1. Whether negative opinion regarding COVID-19 vaccines exists on Twitter.
2. Whether lexicon-based (PYTHON/VADER) and machine learning (Microsoft Azure) approaches to sentiment classification yield different sentiment results.
3. Whether low levels of concern about COVID-19 vaccines lead to high acceptance of the vaccine.
4. Whether public opinion towards COVID-19 vaccinations becomes more positive over time.

## 2. Materials and Methods

### 2.1. Data Collection

In order to share information on Twitter as widely as possible, Twitter provides broad access to public Twitter data via their own Application Programming Interface (API). In this study, Twitter’s official API was used to collect tweets in real time between 1 July 2021 and 21 July 2021. The language filter arguments “EN” and “RT” were applied to only select English tweets and filter out re-tweets. Tweet scraping was conducted using 43 search terms relating to COVID-19 vaccinations (Table 1) on Twitter’s asymmetric cryptography (OAuth2) process and saved into an SQLite database. Following a small pilot study to establish which key words would be most useful to investigate, key words were selected based on the COVID-19 vaccines available in the UK at the time of data collection and also to avoid collecting a large number of tweets that would have discussed vaccines in general rather than being specifically related to COVID.

A total of 137,781 tweets were collected and stored in a database. Data collected included the user’s display name, twitter handle, tweet text and date/time the tweet was published.

### 2.2. Sentiment Data Analysis—Machine Learning Approach (MLP)

Primary sentiment analysis was conducted on the dataset using Azure on Microsoft Excel. The software yielded the results as ‘positive’, ‘negative’ or ‘neutral’ and scored the confidence of the analysis, with a score of 1 being most confident with the analysis and 0 being least confident.

### 2.3. Sentiment Data Analysis—Lexicon-Based Approach

A Python-based API for Twitter was used to collect live tweets, which were recorded into a relational database using SQLite. Sentiment analysis was performed post-collection using the VADER algorithm, as part of the NLTK Python package. It is worth noting that Python version 3.9.0 was used throughout this process. Custom-made software built with Python 3.9.0 was used to perform the word frequency analysis. NLTK was used in the pre-processing of tweets—to remove stop words—prior to the word frequency analysis.

The provided sentiment compound—or sentiment score—calculated from the sum of lexicon ratings, was normalised between −1 (extreme negative) and +1 (extreme positive). This technique determined the polarity—or positivity and negativity—and the intensity of the expressed emotion. The intensity of emotion of each tweet is divided into the quantity of positive, negative and neutral elements the tweet contained—adding to a total value of 1. Each tweet was classified as positive, negative or neutral according to its compound score. Compound scores less than 0.05 were considered negative, scores between −0.05 and 0.05 were considered neutral and scores above 0.05 were classified as positive [41,55].

### 2.4. Statistical Analysis

Descriptive statistics analysed differences between the program outputs and to test for significance between approaches, sentiment frequency, and sentiment against time. Questionnaire results were analysed on JISC (www.jisc.ac.uk, (accessed on 12 August 2021)) [56] automatically. Chi-square tests, two-way ANOVA and descriptive statistics were performed on Microsoft Excel and Statistics Kingdom (www.Statskingdom.com, (accessed on 14 August 2021)) [57] and Welch’s and two-sample *t*-tests were performed using Python 3.9.0 and MATLAB.

### 2.5. Questionnaire

Using the JISC software to design, distribute and record the results, the questionnaire (Table A1)—composed of 22 questions—was distributed to anonymous adult participants (*n* = 182). The questionnaire was designed to investigate attitudes towards COVID-19 disease and COVID-19 vaccinations with the aim to determine personal knowledge and opinion of vaccinations as well as identifying factors that may influence vaccine hesitancy. Demographic data including age (18–29, 30–39, 40–49, 50–59, 60–69, and 70+) and parenthood status were recorded by the respondents. Questions including whether participants have previously received vaccinations for themselves or their children and whether they have accepted or will accept a COVID-19 vaccination were posed. Free-text opportunities to elaborate on the reasons for declining vaccinations for themselves or their children were provided. The participants were also asked agree/disagree-style questions relating to COVID-19 vaccinations and their general knowledge surrounding vaccinations. The questionnaire was distributed via email and social media platforms including Twitter and Facebook. Incomplete responses were excluded from this study.

## 3. Results

### 3.1. Python Sentiment Analysis

#### 3.1.1. Tweet Sentiment Scores

**Figure 1:**
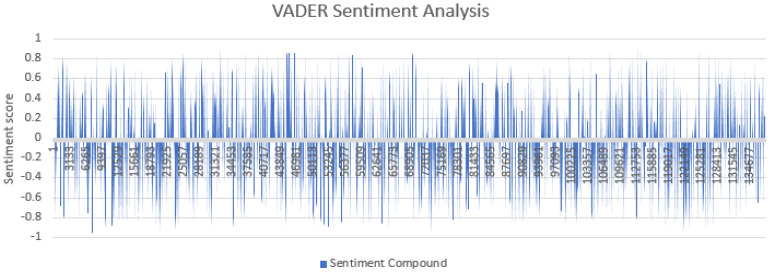
VADER sentiment scores for each tweet. Values greater than 0.05 are displayed as positive, values between −0.05 and 0.05 are neutral and values less than 0.05 are negative tweets. The lengths of the peaks represent the intensity of negativity or positivity. Values represent the tweet number. The horizontal axis shows the tweets in order, ranging from 1 July 2021 (**left** of graph) to 21 July 2021 (**right** of graph).

The VADER algorithm is the gold standard used among sentiment researchers [47]. Due to its wider term coverage [58], quick application [41] and high classification accuracy [59], we opted to use the results from this approach for the rest of this study. Between 1 July 2021 and 21 July 2021, Python scraped a total of 137,781 tweets relating to the chosen search terms. The compound scores were plotted against time (Figure 1). There was no obvious trend from the graphical representation, and therefore sentiment groups were investigated individually.

#### 3.1.2. Word Frequency

**Figure 2:**
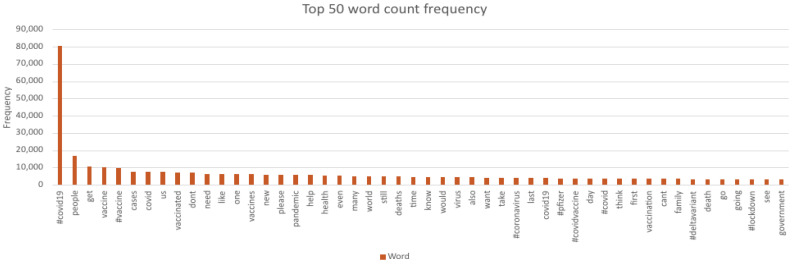
Top 50 frequently recurring words.

The word count (Figure 2) shows the most frequently identified term was clearly ‘#covid19′ with other terms such as ‘people’, ‘get’ and ‘vaccine’ also frequently used. There was no mention of specific groups such as ‘children’ or ‘parents’, only the collective term ‘people’.

**Figure 3:**
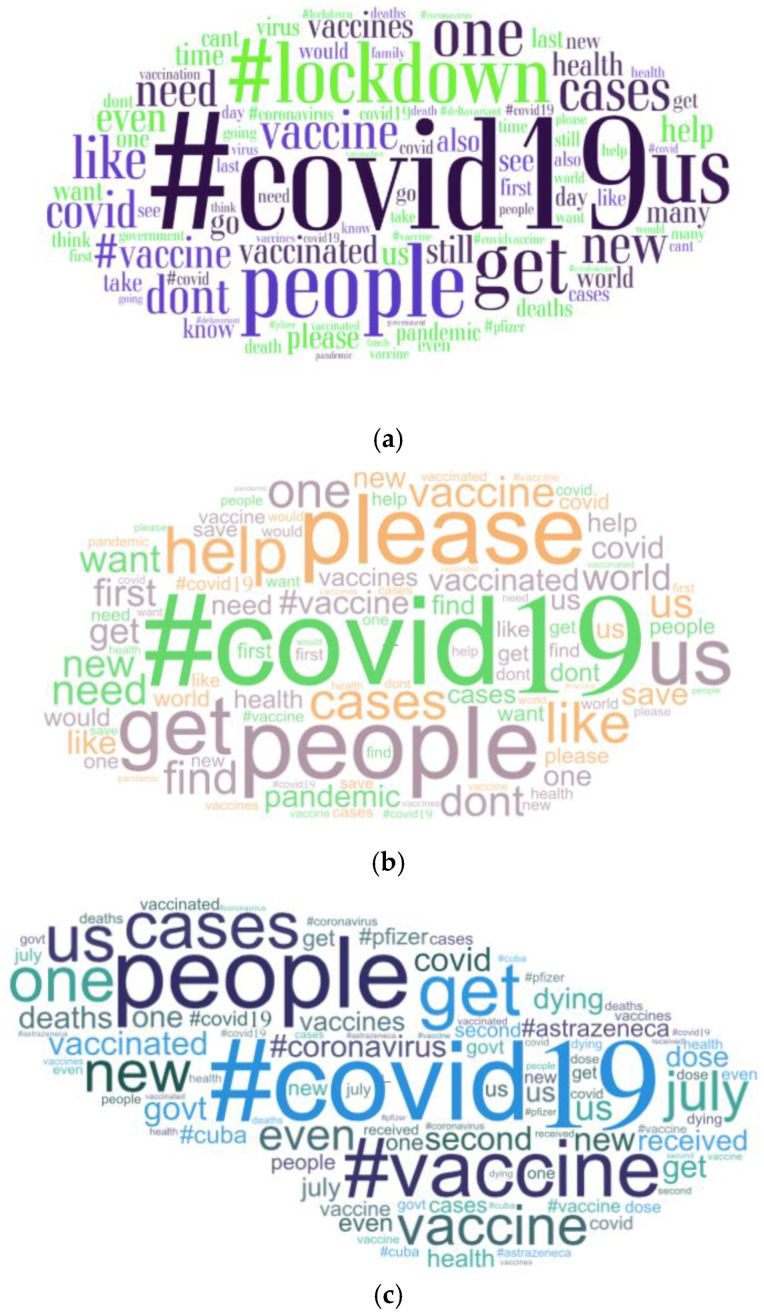

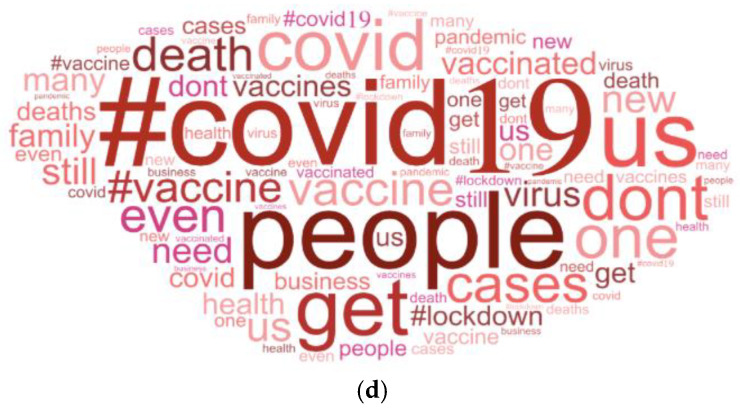
(**a**) Word cloud of the top fifty repeated words (https://wordart.com/, (accessed on 15 August 2021)); (**b**) word cloud of the top twenty-five most repeated words in the positive category; (**c**) word cloud of the top twenty-five most repeated words in the neutral category; (**d**). word cloud of the top twenty-five most repeated words in the negative category.

A word cloud (Figure 3a) displays the most frequently used words in size descending order. The larger-sized words depict a higher frequency of the word. To further understand the relationship between words and their frequency, analysis into the most prevalent words was conducted from the separate positive, negative and neutral groups.

In the positive category (Figure 3b), the most commonly recurring words were ‘#covid19′ (29,661), ‘people’ (5313) and ‘please’ (4455). In the neutral category (Figure 3c), the most commonly used words were ‘#covid19′ (14,399), ‘people’ (2469) and ‘#vaccine’ (2322). In the negative category (Figure 3d), the most commonly used words were ‘#covid19′ (31,725), ‘people’ (7925) and ‘get’ (4282). Noticeable words in this category include ‘don’t’, ‘get’, ‘vaccinated’ and ‘death’, which could suggest that users are advising others not to receive the vaccinations.

The frequency and percentage (Table 2) of the sentiment of tweets in each week were determined to establish whether there was a trend across time between the groups.

**Figure 4:**
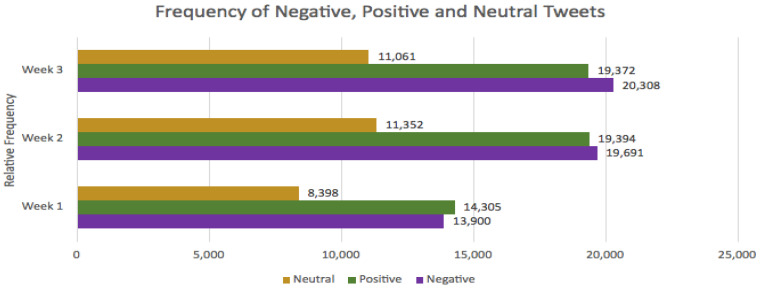
Frequency of negative, positive and neutral tweets over a 3 week period. The frequency of all sentiment groups increased in week 2 compared to week 1. The frequency of negative tweets continued to increase into week 3, whereas positive and neutral tweets slightly decreased.

During week 1, positive tweets were the most frequent (14,305; 39.0%) compared to negative (13,900; 37.9%) and neutral (8398; 22.9%). By week 2 and week 3, negative tweets (19,691; 39.0% and 20,308; 40.0%, respectively) were most frequent compared to positive (19,394; 38.4% and 19,372; 38.1%) and neutral (11,352; 22.5% and 11,061; 21.7%) (Table 2, Figure 4).

**Figure 5:**
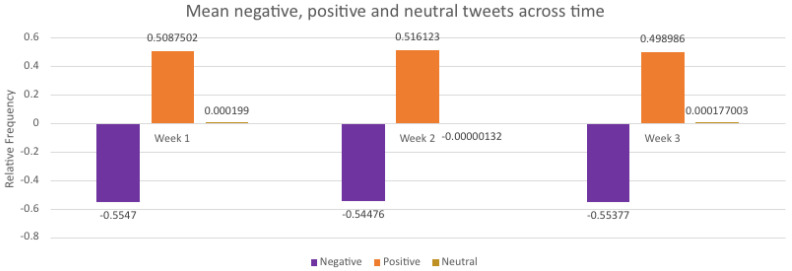
Average values of negative, positive and neutral scores displayed over time. During week 2, the mean values for neutral tweets are lower (>−0.01) than the previous and following week.

To determine whether there was a significant difference between the frequency of positive, negative and neutral scores, mean values were established for each week of data collection (Figure 5).

A two-sample *t*-test with equal standard deviation was performed between the first and final week of each sentiment group to investigate difference over time. The positive average (0.508; SD = 0.511) during week 1 was found to be equal to the positive average in week 3 (0.498; *p* = 0.110). The Test statistic (*t* = 1.597) was found in the 95% critical value accepted range. The negative average (−0.554; SD = 0.511) values during week 1 were found to be equal to the negative average in week 3 (−0.553; *p* = 0.858). The Test statistic (*t* = −0.177) was in the 95% critical value accepted range. The neutral average (0.00019; SD = 0.511) values during week 1 were found to be equal to the negative average in week 3 (0.00017; *p* = 0.997). The Test statistic (*t* = 0.003) was in the 95% critical value accepted range.

#### 3.1.3. Intensity of Sentiment

Week 1 (−0.345, 0.508, 0.00019) and week 3 (−0.358, 0.499, 0.00017) displayed similar trends of negative, positive and neutral tweets, respectively (Figure 5). During week 2, neutral tweets displayed more negativity than positivity (−1.322).

The means of tweets were subjected to a two-way ANOVA (Table 3). The difference between weeks is not statistically significant (*p* = 0.1951), which is indicative of no significant change in mean values between weeks. The difference between averages of the sentiment results (i.e., negative mean value against positive mean value against neutral mean value) is statistically significant (*p* < 0.0001).

Negative tweets had a higher mean value (0.52706) than positive (0.48196) and neutral (0.50119) tweets (Table 4). To compare the means between the groups, Welch’s *t*-test (two-sample *t*-test) was performed (due to unequal variance and differing *n*) using MATLAB. Firstly, the values were normalised by mapping to the range of 0–1, where 0 is the “least” and 1 is the “most”, i.e., negative tweets were mapped from [−1, −0.05] to [0, 1], where 0 is least negative (−0.05) and 1 is most negative (−1). This was achieved using an inverse interpolation function (t−a)/(b−a), where t is the value, a is the lower bound and b is the upper bound.

Welch’s *t*-test demonstrated that positive vs. negative (*p* < 0.001), positive vs. neutral (*p* < 0.001) and negative vs. neutral (*p* < 0.001) groups show statistical significance between the means. This suggests that sentiment across our dataset displays a larger intensity of negative sentiment compared to positive or neutral., i.e., the negative tweets are “more” negative than the positivity in positive tweets.

### 3.2. Machine Learning vs. Lexicon Based: A Comparison of Negative, Positive and Neutral Tweets

The Natural Language Toolkit (or NLTK) (https://www.nltk.org/, (accessed on 21 July 2021)) [60] was used for the VADER sentiment analysis and scored 53,899 tweets as negative, 53,071 as positive and 30,811 as neutral, whereas Azure determined the frequency of the categories as 67,538, 45,282 and 24,961, respectively. They reveal similar trends whereby most tweets were negative, followed by positive and neutral tweets being least prevalent (Table 5).

**Figure 6:**
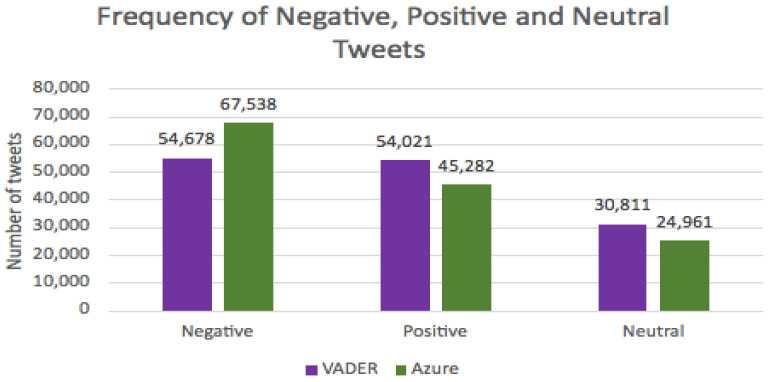
Total number of negative, positive and neutral tweets as determined by Microsoft Azure and VADER.

The lexicon-based (VADER) and machine learning (Microsoft Azure) approaches to classify sentiment were compared (Table 5, Figure 6). A total of 39.11% of tweets were scored as negative by VADER and 49.01% were scored as negative by Azure. The percentage of tweets scored by VADER and Azure as positive were 38.51% and 32.86%, respectively. A total of 22.36% and 18.11% were considered neutral.

### 3.3. Questionnaire

The questionnaire collected a total of 188 responses. A total of 6 responses were excluded due to the participants not meeting the requirements for this study or not agreeing to their data being shared and so we used the complete 182 responses in the analysis (Table A1).

A total of 31.9% of participants were between 18 and 29 years (the largest age group of participants), with 90.1% stating they had previously searched for information regarding COVID-19 online (e.g., Google). The most common length of time spent on social media was recorded as ‘daily’ (64.3%). Most of the participants (85.7%) had previously accepted all vaccines they had been offered), 73.8% were not concerned about receiving a COVID-19 vaccination, 17.1% were slightly concerned, 4.3% were very concerned and 4.3% stated that they were impartial.

We asked whether participants had accepted—or will accept—a COVID-19 vaccine. Of the 182 participants, 8.2% have not/will not accept the vaccine, 1.6% said they did not know, and the majority (90.1%) stated that they had already or would accept a vaccine. The most likely reason (40.2%) for accepting a COVID-19 vaccine was ‘I want the world to go back to how it used to be before the COVID-19 pandemic’, whereas the most common reason for not accepting the COVID-19 vaccine was ‘I have done my own research and do not believe them to be safe’ (52.9%).

**Figure 7:**
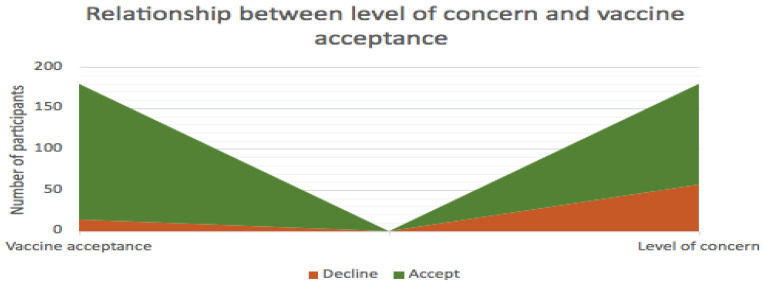
The relationship between level of concern and acceptance and rejection of a COVID-19 vaccine.

In response to whether the participants would allow their child under the age of 18 to have a COVID-19 vaccination if they were offered them in the future, 26.8% would not vaccinate and 5.4% probably would not vaccinate their children against COVID-19. A total of 17.9% were unsure whether they would vaccinate their children, 8.9% probably would and 41.1% said yes, they would vaccinate their children. Participants with adult children (18 or older) or without children automatically skipped this question. We compared level of concern to vaccination acceptance or rejection (Figure 7). Out of 52 participants showing some level of concern, 15 of these participants rejected the vaccine.

We asked how the participants would consider their current depth of knowledge regarding vaccinations generally. Knowledge scores ranged from 0 (no knowledge) to 5 (deep/thorough knowledge). Overall, 2.2% stated that they had no understanding, 74.2% felt they had some understanding, and 23.6% had a deep understanding.

Several chi-square tests (significance level, alpha, of 0.05) were performed to determine whether there was an association between certain vaccine refusal prediction factors (Table 6). The results show that the uptake of COVID-19 vaccines was dependent on previous vaccine history (*p* < 0.001) and an individuals’ level of concern (*p* < 0.001). However, vaccination understanding (*p* = 0.949491), age (*p* = 0.057899) and time spent on social media (*p* = 0.925771) did not influence the acceptance of COVID-19 vaccinations. Chi-square analysis was also performed between responses of the statement ‘Vaccine safety and effectiveness data are often false’ and intensity of concern and found a significant relationship (*p* < 0.001) (Table 6). The majority of respondents who were not concerned about receiving a COVID-19 vaccine ‘strongly disagreed’ with the statement (52.89%), whereas those who were most concerned stated that they ‘don’t know’ (42.86%).

## 4. Discussion

### 4.1. Machine Learning vs. Lexicon-Based Approaches

Sentiment analysis research has become popular over the past two decades [40,61,62]; as more efficient sentiment classification models are devised [63] and studies have compared automated analysis of conversations on social media with manual approaches [64].

Prior studies have compared machine learning methods of text analysis (i.e., SVM) with lexicon-based approaches [28,65,66] and often conclude the machine learning methods are more effective. For example, Sattar et al. (2021) concluded that VADER was less accurate than machine learning applications and used TextBlob in their study [28]. However, Dhaoui et al. (2015) determined that both approaches performed similarly when analysing Facebook reviews for both positive and negative classification [67]. Much of the literature on this is contradictory and highlights the need for continued research in this area of comparing the accuracy and precision of the machine and lexicon methods. For example, Nguyen et al. (2018) stated that SVM displayed 89% accuracy and 90% precision in comparison to VADER (83% and 90%, respectively) [68], whereas in a different study, SVM’s accuracy and precision were different (71.8% and 66.8% and, respectively) as were that of lexicon-based approaches (71.1% and 65.1% and, respectively) [69]. Despite much of the literature claiming the inferiority of lexicon-based approaches, our research required classification of how positive and negative online sentiment was: one advantage of the VADER model [41].

In other studies, Microsoft Azure has been found to yield better results when compared to other analyser tools such as Stanford NLP [64], IBM Watson Natural Language Understanding, OpinionFinder 2.0 and Sentistrength [70]. However, as Azure only identifies polarity, it is a less accurate method of measuring an individual’s opinion towards a topic compared to other approaches such as VADER [71] and so part of this study compared the sentiment analysis approaches of Microsoft Azure and VADER.

Previous studies have explored sentiment surrounding COVID-19 vaccinations on Twitter [72,73]. Xue et al. (2020) used Latent Dirichlet Allocation (LDA)—a machine learning approach—and collected four million tweets on COVID-19 using 25 search words. Their aim was to identify popular themes, sentiment, bigrams and unigrams. The NRC Emotion Lexicon classified sentiments into several emotions including anger, fear, surprise, sadness, disgust, joy, trust and anticipation and revealed that Twitter users display ‘fear’ when discussing new cases of COVID-19, as opposed to ‘trust’ [74]. Bhagat et al. (2020) used TextBlob to perform sentiment analysis and scraped 154 articles from blogging and news websites. Over 90% of the articles were positive and blogs were found to be more positive than newspaper articles [75]. Sattar et al. (2021) adopted a similar approach to the present study, analysing COVID-19 vaccine sentiment using a large number of tweets (*n* = ~1.2 million) using a lexicon-based classifier, namely VADER and TextBlob. They also defined their neutral sentiments between −0.05 and 0.05 and determined that public sentiment was more positive than negative.

### 4.2. Word Identification and Word Frequency

The results confirm that negativity towards the COVID-19 vaccines is present on Twitter alongside tweets that are positive and neutral in sentiment. Similar studies corroborate these results [10,49,76], with suggestions that development speed and safety concerns are some of the reasons why hesitancy is expressed [77]. Chandrasekaran et al. examined the trends of sentiment of several topics associated with COVID-19 between January 2020 and May 2020 and found that although Twitter users expressed negativity about the spread and symptoms of COVID-19, they determined that positive feelings were expressed when sharing information on drugs and new therapies [55]. In the present study, the commonly used term ‘people’ suggests that concerns do not specifically relate to children, elderly or any other specific group. Although the hashtag ‘#covid19′ was the most frequently occurring word in all three sentiment groups, analysis found that a higher number of negative tweets contained the hashtag (31,725) in comparison to positive (29,661) and neutral (14,399) tweets.

A study on the sentiment surrounding human papillomavirus vaccines found different keywords associated within their word clusters. The authors suggested that ‘HPV’ was associated with personal words including ‘I’ and ‘me’ and ‘#HPV’ was associated with words such as ‘learn’ and ‘prevent’. The authors considered these ‘awareness-raising words’ [78]. Our findings show similar results; ‘people’, ‘don’t’, ‘health’, ‘vaccines’, and ‘death’ were noticeable in the negative groups. This could also be indicative of concerns about the risks of accepting the vaccine [79]. Words including ‘people’, ‘please’, ‘help’ ‘vaccine’ ‘first’ and ‘need’ were found to be frequently occurring in the positive group. These terms suggest that discourse leans towards promotion and encouragement of vaccinating, with similar key words found in previous studies [79]. The only similarities of the word frequencies performed by Sattar et al. (2021) and this study were ‘death’ and ‘people’ in the negative category, ‘vaccine’ in the positive category and ‘help’ and ‘first’ in both the positive and neutral categories. They also identified words that were not found in our study including ‘party,’ ‘happy’ and ‘thank’ [28].

Previous research suggests that social media users tend to interact with others who share common beliefs and ignore or argue with individuals who have opposite views [80,81], creating an echo chamber. Due to this, it has been suggested that public health interventions could reinforce vaccine hesitancy [81,82,83] and identifying keywords or hashtags that hesitant individuals commonly use would be a more effective strategy [84] to countering the problem. This study has identified several keywords and hashtags to assist in this process.

### 4.3. Relative Frequency of Tweets

We observed the frequency and relative frequency of tweets in each week of this study. Despite most of the tweets in the dataset being negative, positive tweets (14,305; 39.0%) were the most predominant during the first week of data collection between 1 July 2021 and 7 July 2021 whereas, in the final two weeks, between 8 July 2021 and 21 July 2021, negative tweets (19,691; 39.0% and 20,308; 40%) were most common. Neutral tweets were significantly lower than both negative and positive tweets throughout the entire time of collection (22.9%, 22.5% and 21.7%). Piedrahita-Valdes et al. (2021) performed sentiment analysis on vaccine-hesitant tweets between June 2011 and April 2019 and found neutral tweets were predominant throughout the study, in contrast to the present study. They also found that negative tweets peaked at times and noted that at least one of these peaks coincided with a documentary linking autism to vaccines. Similarly, they identified positive-related peaks occurring in April which coincided with World Immunisation week [25]. Furthermore, a noticeable increase in anti-vaccine discourse was experienced on Twitter in 2015, coinciding with a measles outbreak (2014–2015), a newly released film “Vaxxed” and the publication of the book “Vaccine Whistleblower” [17], supporting the idea that conversations relating to vaccine hesitancy fluctuate over time.

The mean of neutral tweets displayed a negative sentiment compound (−0.00000132) during week 2 of the investigation, whereas, in weeks 1 and 3, neutral tweets were positive (0.000199 and 0.000177, respectively). This is suggestive of concurrent events that the general public are exposed to [17] such as case numbers, the reporting of daily hospitalisation and death figures, the pace of the UK vaccination programme and the expansion of testing capability in addition to wider political factors including legislated social distancing, lockdowns, working from home mandates and face mask wearing. For example, on 5 July 2021 plans to remove the mandated wearing of facemasks from 19 July 2021 were announced in England. This announcement could have been a key factor in the high positive sentiment we detected in this study in week 1. By 7 July 2021, however, the UK’s weekly COVID-19 cases had doubled in comparison to the week prior; and between 8 and 14 July (corresponding to week 2 in this study), cases continued to rise in the UK, with over 50,000 new cases reported on 17 July 2021 [85]. As these events unfolded, 1200 scientists formally challenged the easing of lockdown restrictions in England [86], a discussion that is likely to have added to the negative sentiment at the time. Public opinion remained polarised and by week 3 of our study, we found the highest frequency of tweets which reflected negative sentiment at the same time as the number of tweets that were positive in sentiment increased from week 2 (38.4%) to week 3 (47.6%). Whilst previous research has identified vaccine hesitancy fluctuating over time [17], it would be interesting to compare the dates of specific announcements and wider discussions with daily sentiment analysis to determine whether there is a relationship between the two.

### 4.4. Questionnaire: Vaccine Hesitancy towards COVID-19 Vaccinations

Our study is the only one to date to incorporate a questionnaire alongside the exploration of sentiment analysis on Twitter towards COVID-19 vaccinations. Most respondents (90.1%) had or would accept a COVID-19 vaccine, a view that is in line with conclusions drawn by other studies [87,88] whilst others have reported less public support for COVID-19 vaccinations [89].

The identification of factors that might predict hesitancy towards COVID-19 vaccines was investigated. A positive correlation between intensity of concern regarding vaccines and their uptake was established, suggesting that participants with higher levels of (or more intense) concern are less likely to accept the vaccine, whereas those with low levels (less intense) or no concern are more likely to accept the COVID-19 vaccine.

Additional predictors of vaccine hesitancy were explored by considering whether age, vaccine history, level of vaccine understanding and usage of social media were likely to influence an individual’s decision to take a COVID-19 vaccination. No association was established between vaccine refusal and age, despite the Pew Research Group (2017) finding younger adults (<30 years) were less likely to consider beneficial aspects of the MMR vaccine outweighed the risks, compared to older age groups [90]. The same study found individuals with higher levels of understanding considered the risk of vaccine side effects as low, whereas there was no association found between vaccination understanding and vaccination uptake in our study. Survey research on COVID-19 vaccine hesitancy corroborated our results by also finding no association between age and vaccine refusal [91] although Bendau et al. (2021) did establish an association between vaccine hesitance and concern [92]. Interestingly, 17.2% of respondents in the present study somewhat or strongly agreed that “vaccine safety and effectiveness data are often false”, suggesting a significant proportion of the general public have concerns trusting this information as evidenced previously [9]. Anecdotal evidence from the questionnaire suggests that participants are more likely to write negative comments. This view is supported by the literature where it is understood that negative emotions (such as anger, frustration, sadness and disappointment) motivate individuals to articulate their views [93,94].

Reports suggest that the acceptance of vaccines in emergency situations (such as a pandemic) differs to that of routinely administered vaccines in non-crisis situations [87]. However, contrastingly, public concerns surrounding safety are higher with the uncertainties that come with novel vaccines and new emerging infectious diseases [87,95,96,97]. For example, in the UK, France, Greece, America and Australia, only 17% to 67% of the general public was willing to accept the vaccine for the H1N1 pandemic in 2009 [95,96,97,98,99,100,101,102], highlighting public concern in this area and also likely variable uptake figures. Chaudhri et al. (2021) established the public had a weakly positive sentiment towards receiving a COVID-19 vaccine [73]. Vaccination history has previously been identified as a major predictor of vaccine uptake [95,98,101,103], a view also identified in the present study which established an association between vaccine history and acceptance. Individuals with full previous vaccination history were more likely to accept a COVID-19 vaccine, further confirming the idea of the echo chamber effect.

The present study has confirmed the idea that vaccine compliance remains inconsistent with negative opinions and hesitancy still widespread [91,92] and the inclusion of a questionnaire provided a greater picture of overall sentiment towards vaccines. The questionnaire revealed generally positive sentiment, whereas more negative sentiment was found online, alongside positive and neutral views. The questionnaire revealed that concerns about vaccines typically centred around trust in safety and effectiveness.

### 4.5. Limitations and Further Work

As part of the pilot work for the present study, we manually categorised the sources (Twitter accounts) as ‘personal’, ‘accredited medical’, ‘news’ or ‘government/public health’. It would have been helpful if we could have extended this into the main study to facilitate a better understanding of the most common sources of misinformation. However, with the large dataset in the main study, this was unrealistic, and we seek an automated approach to this for future studies.

The data were collected over a short period in July 2021 and so it would be interesting to extend this study to look at historical and future tweets to further understand whether public opinion regarding COVID-19 vaccinations changed during the course of the pandemic. It would also be interesting to compare the dates of specific events in the media with daily sentiment analysis to determine whether they are closely related.

The questionnaire was distributed via social media and so responses were limited to people with access and were typically in the authors’ extended networks. Future studies should endeavour to distribute the questionnaire more widely and in particular to reach public without access to social media. Concern exists in the UK that certain groups are more susceptible to vaccine misinformation and we would like to reach those communities with future research. This is also the case with the sentiment analysis which only collected tweets in English and therefore had the potential to miss the view of non-English speaking groups in the UK.

A simplified interface would benefit this research as the low accuracy of Microsoft Azure and the complexity of using data mining and analysis tools such as Python requires specific computing expertise. Thus, a simplified graphical interface is in development that would benefit future projects seeking to collect datasets for analysis without a need for an understanding of Python or the VADER algorithm.

Sentiment analysis is a popular and rapidly developing area. An interesting avenue for further research would be to compare our approach using VADER to other language-encoder-based approaches (such as using Bert or GPT), in particular exploring whether these could be useful developments that would work with NLTK.

## 5. Conclusions

This study established that machine learning and lexicon-based sentiment analysis methods yielded different frequencies of sentiment results. Negative sentiment was found to be most frequent online, with a higher intensity of negativity within the neutral tweets. There was no significant change in sentiment towards COVID-19 across the three-week data collection period. Positive correlations were established between COVID-19 vaccine acceptance with full vaccination history and low levels of concern.

Sentiment analysis provides evidence to assess public perception about various topics [104], allowing officials in charge of managing the impact of COVID-19 and health policy makers insight into how the public feel about vaccination safety and efficacy so they can identify areas and misconceptions that need to be addressed [93,94].

The identification of frequently occurring negative terms and of predictors that influence vaccine hesitancy can be utilised to deploy effective strategies such as educational campaigns to increase public confidence in the COVID-19 vaccines and improve vaccine uptake. To ensure vaccination uptake targets are met, this requires continued attention.

## Figures and Tables

**Table 1:** Text mining parameter details.

Parameters	Details
Search terms	Vaccineforall, Vaccine, Antivaccine, Vaccinationcovid, Covid19, AstraZeneca, Astrazenecavaccine, Pfizer, Pfizervaccine, UKvaccinerollout, Covidvaccine, Covidvaccination, Covid19vaccine, Covid19vaccination, Modernavaccine, Oxfordvaccine, UKvaccine, AZvaccine, vaccinesideeffects, Antivax, Antivaxxer, Antivaxxers, OxfordAZvaccine, Moderna, Modernasideffects, Astrazenecasideffects, Pfizersideffects, Oxfordsideffects, seconddose, firstdose, Vaccineconspiracy, UKfightscorona, Covid19UK, Covidenier, vaccinehesitancy, AZvax, modernavax, anti-vaccination, anti-vax, anti-vaxxers, pro-vax, covid19jab

**Table 2:** Frequency and percentages of tweets collected for each week.

Week	Negative Tweets		Positive Tweets		Neutral Tweets		Total Frequency
	Frequency	Percentage (%)	Frequency	Percentage (%)	Frequency	Percentage (%)	
1	13,900	37.9	14,305	39.0	8398	22.9	36,603
2	19,691	39.0	19,394	38.4	11,352	22.5	50,437
3	20,308	40.0	19,372	38.1	11,061	21.7	50,741
Total	53,899		53,071		30,811		

**Table 3:** Descriptive statistics of two-way ANOVA of the mean values of sentiment groups.

Source	DF	Sum of Square (SS)	Mean Square (MS)	F Statistic (df_1_df_2_)	*p*-Value
Week	2	0.0001162	0.00005809	2.528 (2,4)	0.1951
Sentiment Groups	2	1.6833	0.8416	36,625.9271 (2,4)	<0.001
Error	4	0.00009192	0.00002298		
Total	8	1.6835	0.2104		

**Table 4:** Descriptive statistics of collected data, post-normalisation.

Category	n ^1^	Mean	Std. dev ^2^
Positive	53,071	0.48196	0.246031
Negative	53,899	0.52706	0.258930
Neutral	30,812	0.50119	0.066879

^1^ Sample size; ^2^ standard deviation.

**Table 5:** Comparison between Python-based VADER and Microsoft Azure sentiment analysis approaches.

Parameters	VADER	Azure
Positive	53,071	45,282
Negative	53,899	67,538
Neutral	30,811	24,961
Median	0	0.459178
Mean	−0.01978	0.445796
Variance	0.262321	0.071255
Skewness	−0.04129	0.00218
SD ^1^	0.512173	0.266937
Total	137,781	137,781

^1^ Standard deviation.

**Table 6:** Chi-square statistical analysis to determine a dependent association between accepting a COVID-19 and the variables in the table. Vaccine safety (far right column) was analysed against how concerned the participant was.

Parameters	Vaccine Knowledge	Age	Time on Social Media	Vaccine History	Level of Concern	Vaccine Safety
Chi-Square (Observed value)	2.14521	14.25356	3.421087	56.18451	116.8076	54.87902
Chi-Square (Critical value)	9.487729	18.30704	15.50731	9.487729	12.59159	9.487729
DF	6	10	8	4	6	15
*p*-value	0.905871	0.161737	0.905227	<0.001	<0.001	<0.001

## Data Availability

Data available on request due to ethical restrictions. The data presented in this study are available on request from the corresponding author. The data are not publicly available due to conditions of ethical approval.

## Appendix A

**Table A1:** Summary of the raw data from participants’ answers (*n* = 182). Due to the different nature of written response options to certain questions, these have been distinguished with quotation marks.

Question		Responses (%)					
1	What is your age?	18–29 (31.9)	30–39 (17.6)	40–49 (12.1)	50–59 (20.9)	60–69 (13.2)	70+ (4.4)
2	Have you used a search engine (e.g., Google) since January 2020 to search for information about Coronavirus or COVID-19?	Yes (90.1)		No (9.4)		Don’t know (0.6)	
3	How often do you use social media (e.g., Twitter, Instagram, Facebook and Snapchat)	Never (2.7)	Rarely (2.2)	Monthly (0.0)	Weekly (3.8)	Daily (64.3)	More frequently than daily (26.9)
4	Do you believe that information on social media is reliable?	Always reliable (1.1)	Sometimes reliable (70.9)	Rarely reliable (24.2)	Never reliable (2.7)	Don’t know (1.1)	
5	Have you ever tested positive for COVID-19?	Yes (7.7)		No (92.3)		Don’t know (0.0)	
6	As far as you are aware, have you accepted all of the vaccinations you have been invited to (excluding COVID-19) since the age of 18?	Yes I have had all vaccinations I have been invited to (85.7)	I have had some of my vaccinations (8.2)	I have not had any of my vaccinations (2.7)	I have not had vaccinations due to an underlying cause (0.5)	I have decided to opt out of vaccinations (2.7)	Don’t know (0.0)
7	Have you already or are you going to accept a vaccine against COVID-19?	Yes (90.1)		No (8.2)		Don’t know (1.6)	
7a	If you selected don’t know, please specify: (optional)	Response 1: “Too early to be sure of safety.”					
		Response 2: “Not sure if I will have my second vaccine.”					
		Response 3: “I would like to know more long term side effects before committing to being vaccinated.”					
8	Have you received a vaccination to protect you against COVID-19	Yes (98.2)		No (1.8)		Don’t know (0.0)	
9	Which vaccine did you receive?	Pfizer (49.1)	Oxford Astra Zeneca (48.4)	Modern (1.9)	Janssen (Johnson & Johnson) (0.0)	Don’t know (0.6)	Other (0.0)
10	Are you concerned about accepting the COVID-19 vaccine/did you have concerns before receiving the vaccine?	I am not/was not concerned (73.8)	I feel/felt impartial (4.3)	I am/was slightly concerned (17.1)	I am/was very concerned (4.3)	Other (0.6)	
10a	If you selected other, please specify: (optional)	Response 1: “I’m informed about side effects and don’t believe what you see in the news without looking at the actual data. So initially concerned but not after looking into the clotting issue.”					
11	Why did (or why will) you accept the COVID-19 vaccine? (Please select the most likely reason)	I have done my own research and I believe them to be safe (20.7)	I want the world to go back to how it used to be before the COVID-19 pandemic (40.2)	I know of or have lost someone to COVID-19 who did not receive the vaccination in time (5.5)	For protection for myself (27.4)	Other (6.1)	
11a	If you selected other, please specify: (optional)	Response 1: “Mainly to protect others.”					
		Response 2: “For protection of the weak and vulnerable as well as myself.”					
		Response 3: “Family member I care for is vulnerable otherwise I may have declined.”					
		Response 4: “NHS worker.”					
		Response 5: “Protection for my high risk family (mother and father).”					
12	Why did (or why will) you not accept the COVID-19 vaccine? (tick all that apply)	I worry I might get COVID019 (0.0)	I have done my own research and I do not believe them to be safe (52.9)	I worry about the adverse reactions (23.5)	I do not believe the trials have been long enough to ensure accurate results (64.7)	Other (23.5)	
12a	If you selected other, please specify: (optional)	Response 1: “I have had both vaccine doses.”					
		Response 2: “I have an immune system. The majority of people do not need a vaccine for covid 19…. In my opinion. My mother also had a severe adverse reaction to the Astra Zeneca jab and is now suffering high blood pressure.”					
		Response 3: “I’ve had the flu jab—that’s all I needed!”					
		Response 4: “I keep myself fit and healthy, I do not have any medical conditions, I ensure I eat a balanced diet and maintain a normal BMI, I exercise frequently and take my general health very seriously thus I did not feel it necessary to have the vaccine. I felt that pressure from colleagues, family and social media made me feel like I didn’t have a choice. I work in an nhs hospital.”					
13	If you have children, what age are they? (If you have multiple children, please select the age of the youngest)	0–4 years (16.3)	5–10 years (7.6)	11–15 years (4.1)	16–17 years (1.2)	18 years + (32.6)	I do not have children (38.4)
14	As of 1 July 2021 in the UK, children under the age of 18 are not routinely offered a COVID-19 vaccine. If this changed and children were offered the vaccine, would you give permission for your child/children to have the vaccine?	Yes (41.1)	Probably (8.9)	Don’t know (17.9)	Probably not (5.4)	No (26.8)	
15	If you selected no/probably not to the previous question, please tick the most relevant box	They have an underlying disorder that prevents them from having vaccinations (0.0)	I do not trust what is in the vaccine (22.2)	I do not believe that they work (0.0)	I do not want them to suffer possible long term adverse reactions (50.0)	Other (27.8)	
15a	If you selected other, please specify: (optional)	Response 1: “Given that the effects on children of the virus is known and proven to be low on children on balance I don’t think any benefits outweigh the negatives as the vaccine has not been out for long.”					
		Response 2: “Children were never in the at risk group. I believe this experimental poison that’s only approved for EMERGENCY use (e.g., not approved like measles/chicken pox/meningitis) will cause life changing side effects or even death. How many dead children from this vaccine are acceptable? 1? 10? 100? We are vaccinating a population over a disease with a 99.7% survival rate-oh and it’s not even 100% effective!”					
		Response 3: “Covid 19 does not affect children… why would anyone vaccinate a child against something that wouldn’t cause them any harm in the first place?”					
		Response 4: “I would like to see more long term data on infants receiving a vaccine before making my mind.”					
16	Have/would you use Twitter to find out information about COVID-19 or Coronavirus?	Yes (11.5)		No (83.5)		Don’t know (4.9)	
17	I would describe my attitude towards receiving a COVID-19 vaccine as:	Very interested (52.7)	Interested (19.2)	Neutral (12.1)	Uneasy (8.8)	Against it (7.1)	Don’t know (0.0)
18	If friends or family were offered a COVID-19 vaccine I would:	Strongly encourage them (61.0)	Encourage them (19.8)	Not say anything (12.1)	Discourage them (1.6)	Strongly discourage them (3.3)	Don’t know (2.2)
19	Taking a COVID-19 vaccination is:	Extremely important (64.6)	Important (21.5)	Neither important nor unimportant (6.1)	Unimportant (2.2)	Extremely unimportant (2.8)	Don’t know (2.8)
20	Do you consider the COVID-19 vaccine more dangerous than the COVID-19 disease?	Strongly agree (6.6)	Somewhat agree (6.6)	Neither agree nor disagree (7.7)	Somewhat disagree (12.1)	Strongly disagree (64.3)	Don’t know (2.7)
21	Vaccine safety and effectiveness data are often false	Strongly agree (5.0)	Somewhat agree (12.2)	Neither agree nor disagree (16.0)	Somewhat disagree (20.4)	Strongly disagree (40.3)	Don’t know (6.1)
22	How would you describe your general knowledge of vaccinations?	Deep/thorough understanding (23.6)	Some understanding (74.2)	No understanding (2.2)		Don’t know (0.0)	
